# Theoretical Considerations and a Mathematical Model for the Analysis of the Biomechanical Response of Human Keratinized Oral Mucosa

**DOI:** 10.3389/fphys.2016.00364

**Published:** 2016-08-29

**Authors:** Aikaterini Tsaira, Panagiotis Karagiannidis, Margarita Sidira, Spyros Kassavetis, Dimitris Kugiumtzis, Stergios Logothetidis, Olga Naka, Argirios Pissiotis, Konstantinos Michalakis

**Affiliations:** ^1^Department of Prosthodontics, Faculty of Health Sciences, School of Dentistry, Aristotle UniversityThessaloniki, Greece; ^2^Laboratory of Thin Films-Nanosystems and Nanometrology, School of Physics, Aristotle UniversityThessaloniki, Greece; ^3^Department of Engineering, Cambridge Graphene Centre, Cambridge UniversityCambridge, UK; ^4^Department of Electrical and Computer Engineering, School of Engineering, Aristotle UniversityThessaloniki, Greece; ^5^Division of Graduate and Postgraduate Prosthodontics, Tufts University School of Dental MedicineBoston, MA, USA

**Keywords:** human oral keratinized mucosa, atomic force microscopy, oral tissue mechanics, contact mechanics, polynomial model, mathematical model, curve fitting

## Abstract

Removable complete and partial dentures are supported by the residual alveolar ridges consisting of mucosa, submucosa, periosteum, and bone. An understanding of the biomechanical behavior of the oral mucosa is essential in order to improve the denture-bearing foundations for complete and partially edentulous patients. The purpose of this paper was to examine the biomechanical behavior of the soft tissues supporting a removable denture and develop a model for that reason. Keratinized oral mucosa blocks with their underlying bone were harvested from the maxillary palatal area adjacent to the edentulous ridges of a cadaver. The compressive response of the oral mucosa was tested by using atomic force microscopy. The specimens were first scanned in order their topography to be obtained. The mechanical properties of the specimens were tested using a single crystal silicon pyramidal tip, which traversed toward the keratinized oral mucosa specimens. Loading-unloading cycles were registered and four mathematical models were tested using MATLAB to note which one approximates the force-displacement curve as close as possible: a. spherical, b. conical, c. third order polynomial, d. Murphy (fourth order polynomial, non-linear Hertzian based). The third order polynomial model showed the best accuracy in representing the force-displacement data of the tested specimens. A model was developed in order to analyze the biomechanical behavior of the human oral keratinized mucosa and obtain information about its mechanical properties.

## Introduction

Removable complete and partial dentures are supported by the residual alveolar ridges consisting of mucosa, submucosa, periosteum, and bone. This supporting area has been calculated to be 23 cm^2^ for the maxilla, and about 12.25 cm^2^ for the edentulous mandible (Hobkirk and Zarb, 2013). It should be mentioned however that these figures vary, depending on the size of the maxilla or mandible and the amount of resorption after teeth extraction. Nevertheless, these numbers are substantially lower than the supporting mechanism of the teeth—i.e., the periodontal ligament—which is about 45 cm^2^, for each dental arch (Hobkirk and Zarb, 2013).

Besides the smaller area of the denture bearing surface in comparison with that of the teeth, there are some other distinct differences too. These include the involved sensory mechanisms and the anatomical features of each structure. The periodontal ligament is connective tissue with a thickness of 0.15–0.35 mm, consisting of collagen, oxytalan and eulanin fibers, glycosaminoglycans and blood vessels ranging between 4 and 47% of the total tissue volume (Jonas and Riede, 1980; Blaushild et al., 1992; Johnson and Pylypas, 1992; Embery et al., 1995; Sloan and Carter, 1995; Michalakis et al., 2012). The periodontal ligament is organized into six different groups of fibers which are not unidirectionally distributed: 1. transeptal, 2. alveolar crest, 3. horizontal, 4. oblique, 5. apical, and 6. interradicular, which are present only between the roots of multirooted teeth (Carranza, 1990; Lindhe et al., 2003). The existence of many blood vessels into the periodontal ligament and the hemodynamic pressure that these vessels exert affects its biomechanical response (Kristiansen and Heyreaas, 1989; Sims, 1995; Ioi et al., 2002a,b). The biomechanical response of the periodontal ligament to occlusal loads is not clear (Caputo and Standlee, 1987). Three focal hypotheses have been made in the past, in order to describe the way in which the periodontal ligament supports the tooth: (i) the tensional mechanism model, supporting the idea that the fibers have a wavy configuration and consequently load transmission from the tooth to the neighboring alveolar bone occurs through a gradual unfolding of these fibers (Mühlemann, 1951; Picton, 1965, 1969), (ii) the viscoelastic model, considering that tooth movement within the socket is controlled more by the vascular elements than by the fibers (Bien, 1966; Fung, 1973; Natali et al., 2004), (iii) the collagenous thixotropic model, supporting the notion that tooth support is possible because of the periodontal ligament's thixotropic gel properties (Kardos and Simpson, 1979, 1980).

The oral mucosa covering the hard palate and the attached gingiva is termed masticatory mucosa and consists of the stratified squamous epithelium at the surface and the lamina propria which lies deeper. The stratified squamous epithelium consists of four layers, which—from most superficial to deepest—are: (a) stratum corneum, (b) stratum granulosum, (c) stratum spinosum, (d) stratum basale (Nanci, 2013). Lamina propria is connective tissue, which is composed of cells, mainly fibroblasts, and an extracellular matrix, consisting of a ground substance and fibers. Fibroblasts are responsible for the secretion of collagen and other elements of the extracellular matrix. The ground substance of the matrix is composed of glycoproteins, glysosaminoglycans and proteoglycans, while the fibers are mainly collagen, providing tensile strength and flexibility to the tissue and elastic fibers, contributing resiliency. Below the oral mucosa of the attached gingiva and the hard palate lies the mucoperiosteum with dense collagenous connective tissue attaching directly to the periosteum. The mucoperiosteum contains fat and salivary glands (Slavkin and Bavetta, 1972; Newcomb, 1981; Clausen et al., 1983; Meyer et al., 1984; Dahllöf et al., 1986; Bourke et al., 2000). An understanding of the biomechanical behavior of the oral mucosa is essential in order to improve the denture-bearing foundations for complete and partially edentulous patients, by better managing traumatized tissues and giving instructions to patients regarding the time which is required for tissues to recover, after applying occlusal loads during daytime. Furthermore, finite element analysis models studying the deformation of oral mucosa under occlusal loading require use of an equation, which unfortunately is not supplied by the bibliography. Nevertheless, finite element analyses of the oral mucosa have been performed in the past and several material models have been adopted in order mucosal behavior to be interpreted. These include linear elastic, biphasic, multi-phasic elastic, and hyperelastic models (Chen et al., 2015). Additionally, knowledge of oral mucosa biomechanics can be helpful in fabricating dental materials with similar or complementary behavior to that of oral tissues (Saitoh et al., 2010; Hong et al., 2012).

Although, numerous articles have been published on the biomechanics of the periodontal ligament (Kurashima, 1965; Komatsu and Viidik, 1966; Daly et al., 1974; Wills et al., 1976; Atkinson and Ralph, 1977; Wills and Picton, 1978; Dorow et al., 2003; Natali et al., 2003; Bergomi et al., 2010, 2011), the research on the biomechanics of keratinized oral mucosa is scarce (Keilig et al., 2009; Goktas et al., 2011).

The purpose of this study was to examine the biomechanical behavior of the soft tissues supporting a removable complete denture and develop a model for that reason.

## Materials and methods

### Tissue preparation

This *in vitro* study was performed in accordance with the guidelines of the Declaration of Helsinki and the research protocol was approved by the Ethics Committee of the Aristotle University (256/06-07-2011), prior to initiation.

**Figure 1:**
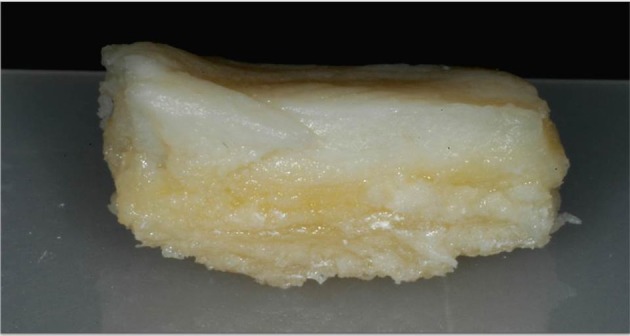
**Human keratinized oral mucosa specimen with the underlying bone, tested with AFM**.

Eight 12 × 8 × 8 mm keratinized oral mucosa blocks with their underlying bone were provided by the Laboratory of Anatomy of the Medical Faculty of the Health Sciences School of the Aristotle University of Thessaloniki (Goktas et al., 2011; Herris et al., 2013). The specimens were harvested from maxillary edentulous areas, by using a low speed 0.2 mm thickness diamond disc (Thin Flex X929-7 TP; Abrasive Technology Inc, Lewis Center, OH, USA) under continuous saline irrigation (Figure 1). To prevent dehydration, the specimens were then stored in a 10% neutral buffered formalin solution (water 91.9–92%, formaldehyde 4%, methyl alcohol 1–2%, sodium phosphate dibasic 0.65%, sodium phosphate monobasic, monohydrate 0.4%) until the mechanical analysis testing, which took place 1 h after.

The compressive response of the oral mucosa was the only biomechanical characteristic tested, by using an Atomic Force Microscope (Solver P47H; NT-MDT Co., Moscow, Russia). A standard square based pyramidal single crystal silicon (Si) tip (NSG 10; NT-MDT Co., Moscow, Russia) with a < 10 nm typical curvature tip radius and a lateral surface of 500 μm was used. A nominal spring constant of 12.9 0.06 N/m was used after calculating it by using the Sader method (Sader et al., 1999). The length of the cantilever was 95 ± 5 μm, the width was 30 ± 3 μm, while the thickness was 2 ± 0.5 μm.

Before initiation of the experimental procedures a calibration of the cantilever took place. First the deflection was converted to force using the Hooke's law and then the response of the cantilever was subtracted from the measurement. For this purpose a force-displacement curve in a hard surface was acquired, followed by a force-displacement curve on the sample. The difference of these measurements supplied the indentation depth.

The specimens were then fixed on glass slabs with Histoacryl topical tissue adhesive (B. Braun Corp., Melsungen, Germany), which was used according to manufacturer's instructions. The fixing luting agent was left to set for a period of 1 min before initiation of the measurements. For each specimen a time-period of 3 min was required for the preparation and testing procedures.

### Specimens topography

The specimens were first scanned in order their topography to be obtained. This can be achieved in two ways: (1) the contact mode and, (2) the tapping (semicontact) mode (Ethier and Simmons, 2008).

**Figure 2:**
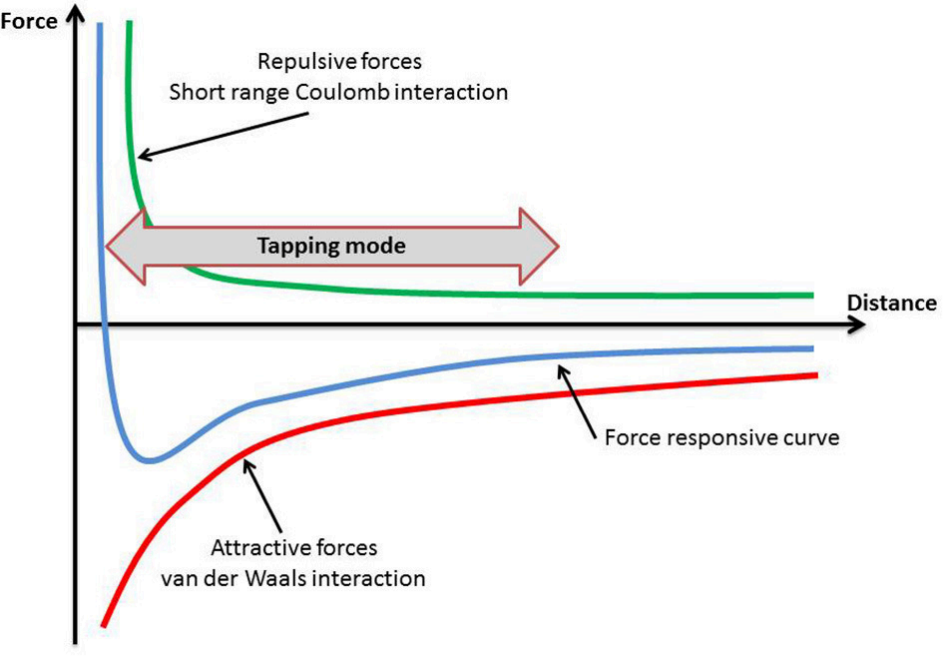
**The tapping mode selected for the purposes of this study**.

In the contact mode the probe exerts a constant force to the specimens, which has as a result the application of large lateral forces and therefore a possible deformation of the specimen (Ethier and Simmons, 2008). Thus, the tapping mode, in which the cantilever is either magnetically or acoustically driven, was selected. (Figure 2) During the scanning period the oscillating tip of the cantilever was moving in very close proximity to the surface of the specimen, touching it regularly.

The oscillations of the cantilever are due to repulsive and attracting forces, and they have been discussed previously in the literature (Goodman and Garcia, 1991; García and San Paulo, 1999). The first ones are short range forces with an exponential decaying and can be considered as Pauli's exclusion principle interaction, electron-electron Coulomb interaction and hard sphere repulsion. The second ones are long range forces, including van der Waals interactions, electrostatic and chemical forces. For the interaction between the tip of the cantilever and the specimen's surface, the van der Waals forces and van der Waals potential obey to the following (Hamaker, 1937):

(1) $$\begin{array}{r} F_{vdW}=-\left(\frac{AR}{{6d}^{2}}\right) \end{array}$$

(2) $$\begin{array}{r} V_{vdW}=-\left(\frac{AR}{6d}\right) \end{array}$$

Where, *A* is the Hamaker constant related to the material, *R* is the sphere radius and *d* is the separation between the tip and the half-space surface (Argento and French, 1996).

The scanning rate was set at ~5,6 μm/s step 11 nm Hz, and the specimens' surface roughness was determined by the two following equations:

Mean absolute deviation surface roughness (*R*a)

(3) $$\begin{array}{l} R\text{a}=\frac{1}{NxNy}\Sigma_{i=1}^{Nx}\Sigma_{j=1}^{Ny}|Z\left(i,j\right)-Z_{mean}| \end{array}$$

Where *Z*_*mean*_ represents the mean height, as this was calculated over the entire area of the biologic specimen, discretized in the grid of *Z(i,j), i* = 1,…,*N*_*x*_ and *j* = 1,…,*N*_*y*_

The Root Mean Square (RMS) surface roughness

(4) $$\begin{array}{l} R_{RMS}=\sqrt{\frac{1}{NxNy}\Sigma_{i=1}^{Nx}\Sigma_{j=1}^{Ny}{\left(Z\left(i,j\right)-Z_{mean}\right)}^{2}} \end{array}$$

which represents the average deviation between the height and the mean surface.

**Figure 3:**
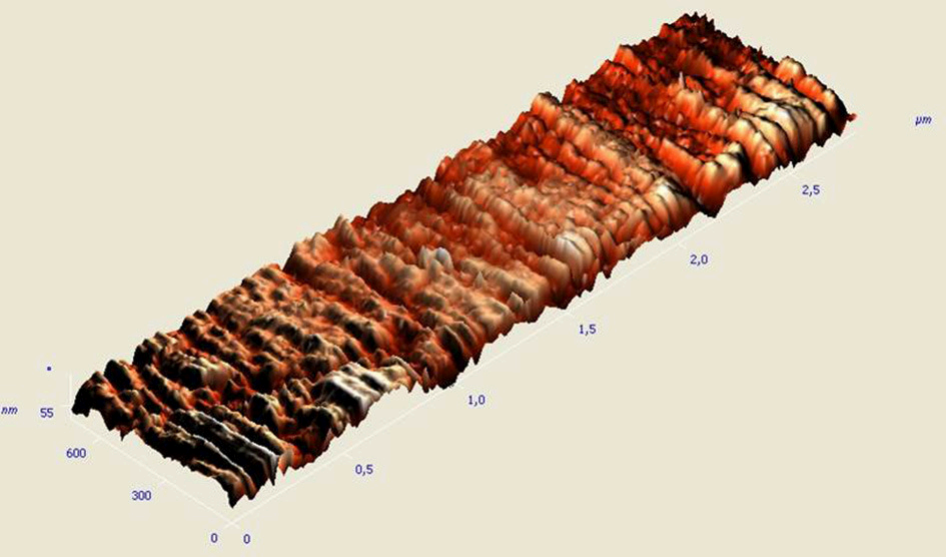
**Three-dimensional AFM image of keratinized oral mucosa**.

Additionally, Ry (peak to valley) measurements were recorded. Two- and three-dimensional images of the specimens' topography were obtained (Figure 3).

### Indentation test

The mechanical properties of the specimens were then tested. The Si pyramidal tip traversed vertically toward the keratinized oral mucosa specimens and the deflection of the cantilever was measured. As the rigid tip moved toward the soft biologic specimen the latter deflected around the probe. This problem of contact mechanics is based on the Hertz theory and the deflection of the cantilever arm is given by the following (Haga et al., 2002; Ethier and Simmons, 2008):

(5) $$\begin{array}{r} z-z_{o}=\frac{F}{k_{c}}+\sqrt{\frac{\pi }{2}\frac{F\left(1-\nu \right)}{E\text{tan}\alpha }} \end{array}$$

**Figure 4:**
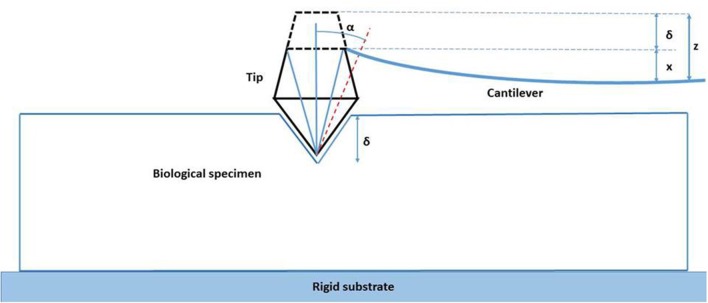
**Indentation of the human keratinized oral mucosa by the Si_**3**_N_**4**_ four-sided pyramid tip**.

Where *z* is the vertical deflection, *z*_*o*_ denotes the height of the probe where the force *F* applied to the biologic specimen becomes non-zero, *k*_*c*_ is the stiffness of the cantilever, *E* is the elastic modulus of the biologic material in N/m^−2^, *v* is the Poisson's ratio of the material (due to high water content Poisson's ratio for most biological specimens is considered to be 0.5), and α is the face angle for the silicon-nitride cantilever (Dimitriadis et al., 2002; Ethier and Simmons, 2008; Figure 4).

The force applied by a four sided pyramidal tip is given by:

(6) $$\begin{array}{r} F=\frac{E\left(\delta \right)}{1-v^{2}}\frac{\tan\;a}{\sqrt{2}}\delta^{2} \end{array}$$

(7) $$\begin{array}{r} \text{while},a=\frac{\tan\;a}{\sqrt{2}}\delta \end{array}$$

**Figure 5:**
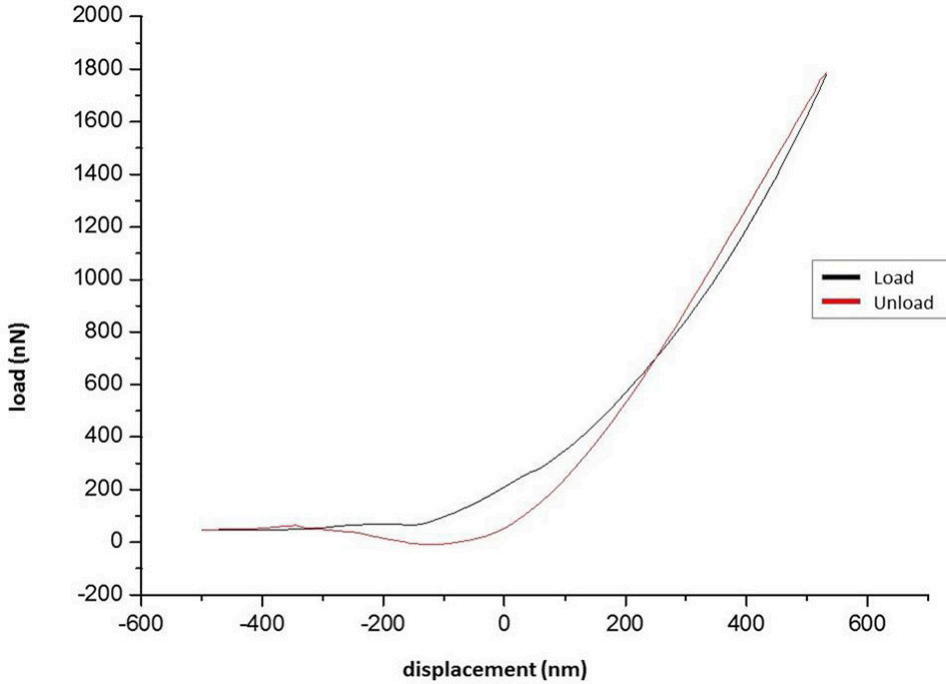
**Load-unload curve**.

A vertical oscillating frequency of ~331 kHz was used and data were recorded at multiple sites of the tested specimens (Weisenhorn et al., 1992, 1993a,b; Stolz et al., 2004; Rahmat and Hubert, 2010; Figure 5). Each loading-unloading cycle lasted 0.3 s.

### Force calibration and displacement data

Identification of the first contact between the tip of the cantilever and the material tested presents a challenge in the indentation tests of soft biologic tissues, when using atomic force microscopy (Stolz et al., 2004; Herris et al., 2013). The abrupt change in the force-development curve, due to the generation of repulsive forces between the two interacting bodies, was taken as the initial contact point (*z*_0_), and the corresponding force (F_0_) at that point was zero.

The indentation depth was given by the following (Rahmat and Hubert, 2010; Herris et al., 2013):

(8) $$\begin{array}{l} h=\left(z-z_{0}\right)-\left(d-d_{0}\right) \end{array}$$

where, *z* denotes the displacement of the cantilever base, *d*_0_ represents the deflection of the cantilever at initial contact and *d* is the deflection of the cantilever. It should be pointed out that the cantilever's deflection was given by

(9) $$\begin{array}{l} d=d_{0}+\left(\frac{F}{k}\right) \end{array}$$

where, *F* represents the force and *k* denotes the stiffness of the cantilever.

Room temperature (21 ± 1°C) and relative humidity (50 ± 10%) were recorded throughout the experiments.

## Results

The indentation tests were performed only on the coronal surface of the specimens. Indentation tests on sagittal planes were not performed due to their low clinical application.

Data from the indentation tests of the tested specimens was collected and force-displacement diagrams have been obtained (Figure 5). It should be mentioned that the negative values in the curve correspond to the initial phase of the testing procedure, when the tip lands on the surface of specimen.

**Figure 6:**
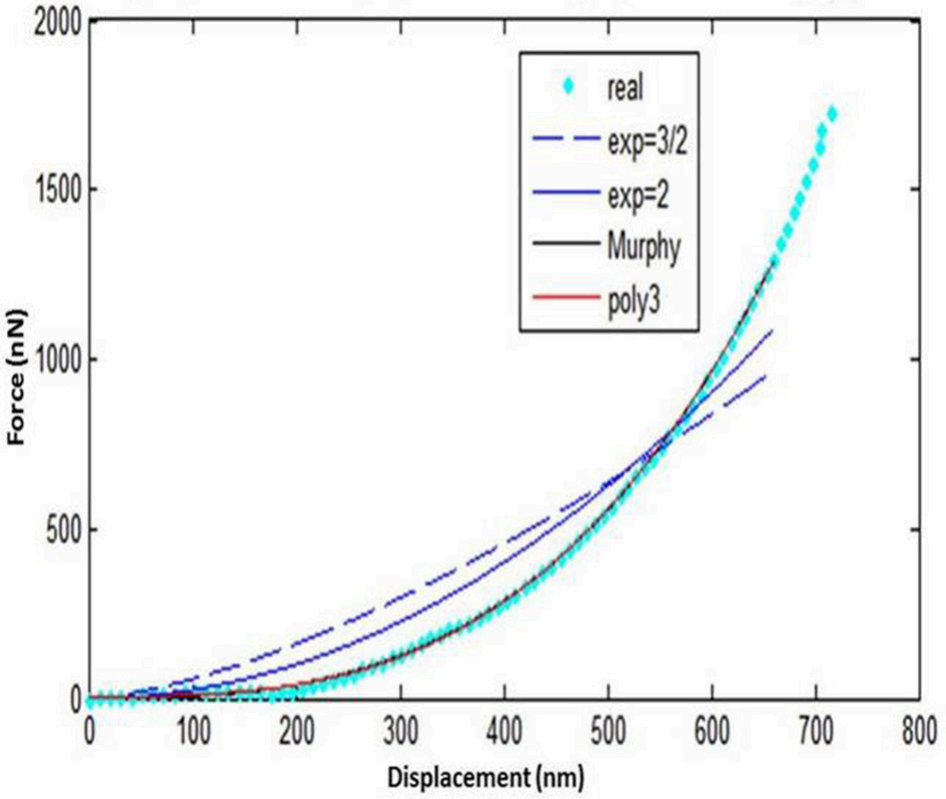
**Oral mucosa force-displacement curve (loading) with different models fitting**.

Five loading-unloading cycles were registered. The simplest fitting approach was selected. That consisted of a visual inspection of the force-displacement curve and identification of the initial contact point (z_o_, d_o_), as already presented earlier (Lin et al., 2007). The area of the curve representing the noncontact region was ignored and for the contact region of the curve four mathematical models were tested using MATLAB (Mathworks Inc; Natick, MA, USA) to note which one approximates the force-displacement curve, as close as possible: a. spherical, b. conical, c. third order polynomial, d. Murphy (fourth order polynomial, non-linear Hertzian based), which is a fourth order polynomial model (Murphy et al., 2013; Table 1, Figure 6).

**Table 1:** **Comparison of the numerical fit results including the goodness-of-fit statistic of the normalized mean square error, where the normalization is by the sample variance**.

**Fit name**	**No.1**	**No.2**	**No.3**	**No.4**	**No.5**
exp = 3/2	0.126403	0.009939	0.021102	0.003426	0.004355
exp = 2	0.047443	0.085310	0.117485	0.060889	0.066963
Murphy	0.000380	0.002366	0.004634	0.002658	0.01629
Polynomial 3	0.000396	0.000071	0.000142	0.000189	0.000023

The goodness-of-fit measure presented in Table 1 is the normalized mean square error (NMSE), and was defined as follows:

(10) $$\begin{array}{l} NMSE=\frac{\sum_{i=1}^{n}{\left(x_{i}-{\hat{x}}_{i}\right)}^{2}}{\sum_{i=1}^{n}{\left(x_{i}-\bar{x}\right)}^{2}} \end{array}$$

where *x*_*i*_ is the *i-th* observation of variable *X*, ${\hat{x}}_{i}$ is the corresponding estimated value by the fitted model, $\bar{x}$ is the sample mean of the *n* observations of *X*.

The spherical and the conical models did not approximate the force-displacement curves The model presented by Murphy et al. (2013), with the form

(11) $$\begin{array}{l} F=\Gamma_{1}\delta^{4}+\Gamma_{2}\delta^{3}+\Gamma_{3}\delta^{2} \end{array}$$

performed well for only one specimen.

The third order polynomial model of the form

(12) $$\begin{array}{l} f\left(\delta \right)=P_{1}\delta^{3}+P_{2}\delta^{2}+P_{3}\delta +P_{4} \end{array}$$

approximated the curve very closely for all tested specimens.

Therefore, a proposition of a model which fits the experimental data better than the aforementioned models and the Hertz model (Herz, 1881), which is fully elastic, is attempted:

Murphy et al. (2013) have speculated that the Young's modulus of biological materials varies with displacement and is given by the following second order polynomial:

(13) $$\begin{array}{l} E\left(\delta \right)=k_{1}\delta^{2}+k_{2}\delta +E_{b} \end{array}$$

The authors hypothesized that the triad set *k*_1_, *k*_2_, and *E*_*b*_ governs the modulus of elasticity of the material they tested. Specifically *k*_1_ and *k*_2_ represent the non-linear region, while *E*_*b*_ represents the contact stiffness, which is in the elastic region of the force-displacement curve.

The coefficients Γ_1_, Γ_2_, and Γ_3_ of their fourth order polynomial (11) are:

(14) $$\begin{array}{r} \Gamma_{1}=\frac{2}{\pi }\frac{k_{1}\left(\tan\;a\right)}{\left(1-\nu^{2}\right)}, \end{array}$$

(15) $$\begin{array}{r} \Gamma_{2}=\frac{2}{\pi }\frac{k_{2}\left(\tan\;a\right)}{\left(1-\nu^{2}\right)},\text{ and} \end{array}$$

(16) $$\begin{array}{r} \Gamma_{3}=\frac{2}{\pi }\frac{{\text{E}}_{b}\left(\tan\;a\right)}{\left(1-\nu^{2}\right)} \end{array}$$

Accordingly, in the present study *E(*δ*)* is given by the following equation:

(17) $$\begin{array}{l} E\left(\delta \right)=k_{1}\delta +k_{2}+E_{b} \end{array}$$

where *k*_1_*, k*_2_, and *E*_*b*_ describe the oral mucosa's and mucoperiosteum's properties as a triad set, and

(18) $$\begin{array}{l} E_{b}=\frac{k_{3}}{\delta } \end{array}$$

*E*_*b*_ represents the mucoperiosteum's contact stiffness and δ is the indentation depth.

A similar approach has been adopted by Murphy et al. (2013). Other researchers have come independently to the same conclusion (Herris et al., 2013).

The constant term P_4_ of (12) is omitted, as when *F* = 0, δ = 0.

Then the general polynomial model (12) becomes:

(19) $$\begin{array}{l} F=P_{1}\delta^{3}+P_{2}\delta^{2}+P_{3}\delta \end{array}$$

Therefore, by substituting *E(*δ*)* from (17) and (18) to (6), the latter becomes:

(20) $$\begin{array}{l} F=\frac{k_{1}\tan\;a}{\left(1-\nu^{2}\right)\sqrt{2}}\delta^{3}+\frac{k_{2}\tan\;a}{\left(1-\nu^{2}\right)\sqrt{2}}\delta^{2}+\frac{k_{3}\tan\;a}{\left(1-\nu^{2}\right)\sqrt{2}}\delta \end{array}$$

where *F* is the applied force, δ is the indentation depth, α is the face angle for the silicon-nitride cantilever, *v* is the Poisson's ratio of the mucosa, and *k*_1_*, k*_2_, and *k*_3_ are the moduli of elasticity for the stratified squamous epithelium, lamina propria and mucoperiosteum respectively.

## Discussion

In the present *ex vivo* study AFM was used to test the mechanical properties of human keratinized oral mucosa. The specimens were harvested from the edentulous areas which support the maxillary complete denture (Hobkirk and Zarb, 2013). The force-development curves were analyzed and a third order polynomial model different than the classic elastic Hertz model (Herz, 1881) was developed in order to describe its biomechanical response.

In the past 25 years AFM has been used to study the mechanical properties of materials. This concept has also been used in the early 90's for “force-displacement” data collection from biological materials, including tendons, ligaments, muscle tissues and menisci (Burnham and Colton, 1989; Radmacher et al., 1992; Tao et al., 1992; Weiss et al., 2002; Sweigart et al., 2004; Yin and Elliot, 2004; Van Loocke et al., 2006, 2008; Villegas et al., 2007; Cheng and Gan, 2008; Ciarletta et al., 2008). A nano-indenter could have been used in this study, as well. However, nano-indenters have a resolution of approximately 100 nN, while the forces applied by the AFM can range from pico-Newtons to several hundreds of micro-Newtons by changing the stiffness of the cantilever. Thus, the sensitivity and the versatility of the AFM makes it an ideal tool for mechanical properties testing of biological materials (Stolz et al., 2009; Notbohm et al., 2012). Additionally, use of the tapping mode of the AFM prevents the distortion of the biologic specimen, as it is presented later in the discussion. In most of the published research, standard manufacturers' cantilevers and pyramidal tips have been employed, while modifications with attachment of silica microspheres have also been reported (Mahaffy et al., 2000; Dimitriadis et al., 2002). The solution for the microsphere tips is supplied by the original Hertz model which deals with the shallow contact between two spherical bodies (Herz, 1881). The original Hertzian theory has been used by many researchers who studied contact deformation and many modifications have been made in order to account for large deformations (Gao and Gao, 2000), viscoelasticity (Gillies et al., 2002; Yang et al., 2004; Attard, 2007; Chen et al., 2013), anisotropy (Batra and Jiang, 2008), multi-layered structures (Ai et al., 2002), and adhesive interactions (Cao et al., 2005; Yang, 2006; Ebenstein, 2011; Chen et al., 2013; Kohn and Ebenstein, 2013). In the present study a sharp pyramidal tip was employed. It should be mentioned that the Bilodeau solution applies for this case (Bilodeau, 1992).

The model developed in the present study is probably valid only when the forces are applied to the coronal part of the oral keratinized mucosa, as it has been proven that tissues are both inhomogeneous and anisotropic. Biological specimens' anisotropic properties have been demonstrated with nonlinear laser scanning microscopy, which has been used for elastin and collagen distribution—within the specimens—imaging (Herris et al., 2013). Additionally their mechanical properties are site and direction-specific dependent (Stolz et al., 2004). It has been shown in the past that the indentation elastic moduli of biological specimens differ depending on the depth, increasing from the superficial to deep layers (Herris et al., 2013). Furthermore, it has been demonstrated that both the Young's modulus and Poisson's ratio change during different development stages of the cell (Zhang et al., 2009). Three different moduli of elasticity were incorporated to the model developed in this study, with k_1_ being the modulus of elasticity of stratified squamous epithelium, k_2_ of lamina propria and k_3_ of mucoperiosteum. This is in accordance with the present experimental results and previous research findings, which have demonstrated that the elastic moduli increase from the superficial to the deep layer (Herris et al., 2013; Murphy et al., 2013). Furthermore, the cells and the extracellular matrix present different elastic modulus and may contribute to the observed inhomogeneity (Trickey et al., 2006; Han et al., 2011). Third-degree polynomial models have also proven to be valid for mechanical forces acting on cell biomembranes, as well as in other anatomical structures as the descending aorta (Stefanadis et al., 2000; Zhang et al., 2009). The third degree polynomial model without a constant term which was tested in the present study not only approximated better the force-displacement curve of the keratinized oral mucosa, but it also provided a simpler model than the fourth order polynomial presented by Murphy et al. (2013). It should be mentioned however that different biological tissues have been examined in these two studies.

As already mentioned, before testing the specimens were kept in a buffered 10% formalin solution until testing, which occurred within an hour. The 10% formalin solution is actually a 4% formaldehyde, which is by definition 1.3 molar. A totally unbuffered formaldehyde solution exerts an osmotic pressure of about 1300 mO. Isotonic salt solutions present osmolarities of 250–350 mO. Therefore, it is expected that formalin diffuses into tissues faster. Formaldehyde has a molecular weight of 30 and it is expected to penetrate the tissues fast. Nevertheless, fixation actually takes a relatively long time. It has been estimated that a time period of 6–16 h, depending on the specimen, is needed. Furthermore, since all specimens received the same treatment simultaneously it can be assumed that formalin penetration was uniform in all specimens. Macroscopically, no swelling was noticed within the 1-h period between specimen harvesting and testing (Bono et al., 2001; Thavarajah et al., 2012).

The human oral keratinized tissue tested in the present study was bonded with cyanoacrylate cement to a glass slab, since there is scientific evidence that force-displacement results depend strongly on whether or not the specimens are attached to the substrate (Yang, 1998). A glass substrate was used as its modulus of elasticity is 50 GPa, which is much higher than that of biologic specimens, while the modulus of elasticity of the pyramidal tip employed was 150 GPa (Grafström et al., 1993; Weisenhorn et al., 1993a). Therefore, the deformation of both the tip and the substrate can be considered as negligible.

The loading-unloading curves of the specimens tested did not coincide, suggesting a viscoelastic behavior (Lakes, 1999). Furthermore, the hysteresis of the loading-unloading curves, indicates dissipation of energy. During loading, the area beneath represents the energy stored, while during unloading, this area represents the energy recovered. Quantification of the hysteresis can be performed by introduction of the plasticity index η. In experiments involving solid objects this parameter characterizes the elastic/plastic behavior of the material, when external forces are applied. The value of plasticity index can range between 0, indicating a fully elastic material, and 1, when the material displays a fully plastic behavior (Briscoe et al., 1998; Klymenko et al., 2009).

The tapping mode was selected for the present study, as it presents more advantages than the contact and the non-contact imaging methods. The tapping mode eliminates the lateral, frictional forces transmitted from the tip of the cantilever to the specimen's surface. In this mode the probe oscillates with sufficient amplitude to prevent it being trapped by adhesive meniscus forces from the contaminant layer (e.g., water), and it makes intermittent contact with the surface of the specimen (Manning et al., 2003; Rogers et al., 2004). As a result, tissue damage is minimal, if any, and recovery is guaranteed. The oscillation frequency usually ranges between 50,000 and 500,000 cycles per second. An oscillation frequency of 311,000 cycles was used in the present study. With this frequency the tip-specimen adhesion forces are overcome and only vertical forces are applied. The high frequency oscillations of the tip may be regarded as a disadvantage of the method, as they are not comparable to the human chewing frequency. It should be mentioned however that, neither the forces applied by the tip to the specimen are comparable to mastication forces. Atomic force microscopy, like all laboratory techniques, has some limitations.

It should be pointed out however that, in studies like the present one, a fundamental assumption is made: the mechanical response of biological materials relies on contribution of different structures which act in sequence. In this way interpretation of the mechanical properties of materials which present a hierarchy can be made (Bonilla et al., 2015).

Development of a model describing the behavior of oral mucosa under mechanical forces is instrumental for the knowledge of its mechanical properties, such as the Young's modulus, and an understanding of how masticatory function is affected by mechanical interactions. Moreover, this modeling assists in fabricating biomaterials (e.g., tissue conditioners) which will act in a similar way with, or in a complimentary way to the oral keratinized mucosa.

It should be mentioned that *ex-vivo* studies performed with AFM present certain drawbacks, including the identification of the most appropriate area for force application, the high frequency oscillation of the cantilever, the possible minor dehydration of the tissue, the molecular roughness of the pyramidal tip and the uncertainty of the first contact between the tip and the biological specimen. These have also been acknowledged by other authors and possibly affect measurement's accuracy (Vinckier and Semenza, 1998).

Further studies are needed to determine the contribution of each layer, as well as, that of the cells and the extracellular matrix in the biomechanical behavior of the oral mucosa.

## Conclusion

Within the limitations of the present study, the following conclusions can be made regarding the force-displacement data of the human oral keratinized mucosa:

1. The third order polynomial model examined in the present study showed a very good accuracy.
2. The Murphy model (fourth order) performed well for only one specimen.
3. The spherical and the conical models did not approximate the force-displacement curves.
4. A mathematical model for the analysis of the biomechanical response of human keratinized oral mucosa was developed.

## Author note

The results of this paper were presented at the IADR General Session, March 20–23, 2013, Seattle, WA, USA, and at the European Society of Biomechanics, August 25–28, 2013, Patras, Greece.

## Author contributions

All authors (AT, PK, MS, SK, DK, SL, ON, AP, and KM) have made substantial contributions to the conception and/or design of the work; or the acquisition, analysis, or interpretation of data for the work; and greatly assisted in drafting the work or revising it critically for important intellectual content; and they approved the submitted version; and agreed to be accountable for all aspects of the work in ensuring that questions related to the accuracy or integrity of any part of the work are appropriately investigated and resolved.

### Conflict of interest statement

The authors declare that the research was conducted in the absence of any commercial or financial relationships that could be construed as a potential conflict of interest.

## References

[B1] Ai Z. Y. Yue Z. Q. Tham L. G. Yang M. (2002). Extended Sneddon and Muki solutions for multilayered elastic materials. Int. J. Eng. Sci. 40, 1453–1483. 10.1016/S0020-7225(02)00022-8

[B2] Argento C. French R. H. (1996). Parametric tip model and force-distance relation for Hamaker constant determination from atomic force microscopy. J. Appl. Phys. 80, 6081–6090. 10.1063/1.363680

[B3] Atkinson H. F. Ralph W. J. (1977). *In vitro* strength of the human periodontal ligament. J. Dent. Res. 56, 48–52. 10.1177/00220345770560011001 264865

[B4] Attard P. (2007). Measurement and interpretation of elastic and viscoelastic properties with the atomic force microscope. J. Phys. Condens. Matt. 19, 47320161 10.1088/0953-8984/19/47/473201

[B5] Batra R. C. Jiang W. (2008). Analytical solution of the contact problem of a rigid indenterand an anisotropic linear elastic layer. Int. J. Solids Struct. 45, 5814–5830. 10.1016/j.ijsolstr.2008.06.016

[B6] Bergomi M. Cugnomi J. Botsis J. Belser U. C. Wiskott H. W. (2010). The role of the fluid phase in the viscous response of bovine periodontal ligament. J. Biomech. 43, 1146–1152. 10.1016/j.jbiomech.2009.12.020 20185135

[B7] Bergomi M. Cugnomi J. Galli M. Botsis J. Belser U. C. Wiskot H. W. (2011). Hydro-mechanical coupling in the periodontal ligament: a porohyperelastic finite element model. J. Biomech. 44, 34–38 10.1016/j.jbiomech.2010.08.019 20825940

[B8] Bien S. M. (1966). Hydrodynamic damping of tooth movement. J. Dent. Res. 45, 907–914. 10.1177/00220345660450036701 5222494

[B9] Bilodeau G. (1992). Regular pyramid punch problem. J Appl Mech 59, 519–523. 10.1115/1.2893754

[B10] Blaushild N. Mihaeli Y. Steigman S. (1992). Histomorphometric study of the periodontal vasculature of the rat incisor. J. Dent. Res. 71, 1908–1912. 10.1177/00220345920710121001 1452892

[B11] Bonilla M. R. Stokes J. R. Gidley M. J. Yakubov G. E. (2015). Interpreting atomic force microscopy nanoindentation of hierarchical biological materials using multi-regime analysis. Soft Matt. 11, 1281–1292. 10.1039/C4SM02440K 25569139

[B12] Bono C. Renard R. Sabatino C. Levine R. Tornetta P. (2001). The effects of varied concentrations of formalin on the tensile strength of cortical bone: should embalmed bone ever be used for biomechanical testing, in Orthopedics Research Society, 47th Annual Meeting February (San Francisco, CA).

[B13] Bourke K. A. Haasse H. Li H. Daley T. Bartold P. M. (2000). Distribution and synthesis of elastin in porcine gingiva and alveolar mucosa. J. Periodont. Res. 35, 361–368. 10.1034/j.1600-0765.2000.035006361.x 11144409

[B14] Briscoe B. J. Fiori L. Pelillo E. (1998). Nano-indentation of polymeric surfaces. J. Phys. D Appl. Phys. 31, 2395–2405. 10.1088/0022-3727/31/19/006

[B15] Burnham N. A. Colton R. J. (1989). Measuring the nanomechanical properties and surface forces of materials using the AFM. J. Vac. Sci. Technol. 7, 2906–2913. 10.1116/1.576168

[B16] Cao Y. F. Yang D. H. Soboyejoy W. (2005). Nanoindentation method for determining the initial contact and adhesion characteristics of soft polydimethylsiloxane. J. Mater. Res. 20, 2004–2011. 10.1557/JMR.2005.0256

[B17] Caputo A. A. Standlee J. P. (1987). Biomechanics in Clinical Dentistry. Chicago, IL: Quintessence.

[B18] Carranza F. A. (1990). Glickman's Clinical Periodontology. Philadelphia, PA: W. B. Saunders.

[B19] Chen J. Ahmad R. Li W. Swain M. Li Q. (2015). Biomechanics of oral mucosa. J. R. Interface 12:20150325. 10.1098/rsif.2015.0325 26224566 PMC4535403

[B20] Chen Z. Diebels S. Peter N. J. Schneider A. S. (2013). Identification of finite viscoelasticity and adhesion effects in nanoindentation of a soft polymer by inverse method. Comput. Mater. Sci. 72, 127–139. 10.1016/j.commatsci.2013.01.040

[B21] Cheng T. Gan R. Z. (2008). Mechanical properties of anterior malleolar ligament from experimental measurement and material modelling analysis. Biomech. Model. Mechanobiol. 7, 387–394. 10.1007/s10237-007-0094-x 17710457 PMC8040535

[B22] Ciarletta P. Dario P. Micera S. (2008). Pseudo-hyperelastic model of tendon hysteresis from adaptive recruitment of collagen type I fibrils. Biomaterials 29, 764–770. 10.1016/j.biomaterials.2007.10.020 17997481

[B23] Clausen H. Vedtofte P. Moe D. Dabelsteen E. (1983). Keratin pattern in human and buccal and hard palate mucosa. Scand. J. Dent. Res. 91, 411–413. 10.1111/j.1600-0722.1983.tb00838.x 6195727

[B24] Dahllöf G. Mondèer T. Reinholt F. P. Wilkström B. Hjerpe A. (1986). Proteoglycans and glycosaminoglycans in phenytoin –induced gingival overgrowth. J. Periodont. Res. 21, 13–21 10.1111/j.1600-0765.1986.tb01432.x 2937892

[B25] Daly C. H. Nicholls J. I. Kydd W. L. Nansen P. D. (1974). The response of the human periodontal ligament to torsional loading- I. Experimental methods. J. Biomech. 7, 517–522. 10.1016/0021-9290(74)90086-4 4452677

[B26] Dimitriadis E. K. Horkay F. Maresca J. Kachar B. Chadwick R. S. (2002). Determination of elastic moduli of thin layers of soft material using the atomic force microscope. Biophys. J. 82, 2798–2810. 10.1016/S0006-3495(02)75620-8 11964265 PMC1302067

[B27] Dorow C. Kristin N. Sander F. G. (2003). Determination of the mechanical properties of the periodontal ligament in a uniaxial tensional experiment. J. Orofac. Orthop. 64, 100–107. 10.1007/s00056-003-0225-7 12649706

[B28] Ebenstein D. M. (2011). Nano-JKR force curve method overcomes challenges of surface detection and adhesion for nanoindentation of a compliant polymer in air and water. J. Mater. Res. 26, 1026–1035. 10.1557/jmr.2011.42

[B29] Embery G. Waddington R. Hall R. (1995). The ground substance of the periodontal ligament, in The Periodontal Ligament in Health and Disease, eds Berkovitz B. K. B. Moxham B. J. Newman H. N. (London: Mosby-Wolfe), 83–106.

[B30] Ethier C. R. Simmons C. A. (2008). Introductory Biomechanics. From Cells to Organisms. Cambridge: University Press.

[B31] Fung Y. C. (1973). Biorheology of soft tissues. Biorheology 10, 139–155. 4728631 10.3233/bir-1973-10208

[B32] Gao Y. C. Gao T. J. (2000). Large deformation contact of a rubber notch with a rigid wedge. Int. J. Solids Struct. 37, 4319–4334. 10.1016/S0020-7683(99)00191-2

[B33] García R. San Paulo A. (1999). Attractive and repulsive tip-sample interaction regimes in tapping-mode atomic force microscopy. Phys. Rev. B. 60, 4961–4967. 10.1103/PhysRevB.60.4961 PMC1300757 10692344

[B34] Gillies G. Prestidge C. A. Attard P. (2002). An AFM study of the deformation and nanorheology of cross-linked PDMS droplets. Langmuir 18, 1674–1679. 10.1021/la011461g

[B35] Goktas S. Dmytryk J. J. McFetridge P. S. (2011). Biomechanical behavior of oral soft tissues. J. Periodontol. 82, 1178–1186. 10.1902/jop.2011.100573 21309720

[B36] Goodman F. O. Garcia N. (1991). Roles of the attractive and repulsive forces in atomic-force microscopy. Phys. Rev. B. 43, 4728–4731. 10.1103/PhysRevB.43.4728 9997840

[B37] Grafström S. Neitzert M. Hagen T. Ackermann J. Neuman R. Ptobst O. (1993). The role of topography and friction for the image contrast in lateral force microscopy. Nanotechnology 4, 143–151. 10.1088/0957-4484/4/3/003

[B38] Haga H. Sasaki S. Kawabata K. Ito E. Ushiki T. Sambongi T. (2002). Elasticity mapping of living fibroblasts by AFM and immunofluresence observation of the cytoskeleton. Ultramicroscopy 82, 253–258. 10.1016/S0304-3991(99)00157-6 10741677

[B39] Hamaker H. C. (1937). The London-van der Waals attraction between spherical particles. Physica 4, 1058–1072. 10.1016/S0031-8914(37)80203-7

[B40] Han L. Grodzinsky A. J. Ortiz C. (2011). Nanomechanics of the cartilage extracellular matrix. Annu. Rev. Mater. Res. 41, 133–168. 10.1146/annurev-matsci-062910-100431 22792042 PMC3392687

[B41] Herris H. K. Miri A. K. Tripathy U. Barthelat F. Mongeau L. (2013). Indentation of poroviscoleastic vocal fold tissue using an atomic force microscope. J. Mech. Behav. Biomed. Mater. 28, 383–392. 10.1016/j.jmbbm.2013.05.026 23829979 PMC3833877

[B42] Herz H. (1881). Über die berührung fester elastischer körper. J. die reine und Angewandte Mathematik 92, 156–171.

[B43] Hobkirk J. A. Zarb G. (2013). The edentulous state, in Prosthodontic Treatment for Edentulous Patients. Complete Dentures and Implant-Supported Prostheses, 13th Edn., eds Zarb G. Hobkirk J. A. Eckert S. E. Jacob R. F. (St. Louis, MO: Elsevier Mosby), 1–27.

[B44] Hong G. Maeda T. Murata H. Sasaki K. (2012). The dynamic viscoelasticity and plasticizer leachability of tissue conditioners. Gerodontology 29, 284–291. 10.1111/j.1741-2358.2012.00639.x 22698258

[B45] Ioi H. Morishita T. Nakata S. Nakashima A. Nanda R. S. (2002a). Evaluation of physiological tooth movements within clinically normal periodontal tissues by means of periodontal pulsation measurements. J. Periodont. Res. 37, 110–117. 10.1034/j.1600-0765.2001.00665.x 12009180

[B46] Ioi H. Nakata S. Nakashima A. Counts A. L. Nanda R. S. (2002b). Changes in tooth position in humans in relation to arterial blood pressure. Arch. Oral. Biol. 47, 219–226. 10.1016/S0003-9969(01)00110-8 11839358

[B47] Johnson R. B. Pylypas S. P. (1992). A re-evaluation of the distribution of the elastic meshwork within the periodontal ligament of the mouse. J. Periodont. Res. 27, 239–249. 10.1111/j.1600-0765.1992.tb01674.x 1640346

[B48] Jonas I. E. Riede U. N. (1980). Reaction of oxytalan fibers in human periodontium to mechanical stress. A combined histochemical and morphometric analysis. J. Histochem. Cytochem. 28, 211–216. 10.1177/28.3.7354216 7354216

[B49] Kardos T. B. Simpson L. O. (1979). A theoretical consideration of the periodontal membrane as a collagenous thixotropic system and its relationship to tooth eruption. J. Periodont. Res. 14, 444–451. 10.1111/j.1600-0765.1979.tb00243.x 161787

[B50] Kardos T. B. Simpson L. O. (1980). A new periodontal membrane biology based on thixotropic concepts. Am. J. Orthod. 77, 508–515. 10.1016/0002-9416(80)90130-X 6154419

[B51] Keilig L. Stark H. Bayer S. Utz K. H. Strazza M. Grünner M.. (2009). Numerical investigation of the mechanical loading of supporting soft tissue for partial dentures. Int. J. Prosthodont. 22, 201–203. 19418869

[B52] Klymenko O. Wiltowska-Zuber J. Lekka M. Kwiatek W. M. (2009). Energy dissipation in the AFM elasticity measurements. Acta Phys. Pol. A. 115, 548–551. 10.12693/APhysPolA.115.548

[B53] Kohn J. C. Ebenstein D. M. (2013). Eliminating adhesion errors in nanoindentation of compliant polymers and hydrogels. J. Mech. Behav. Biomed. Mater. 20, 316–326. 10.1016/j.jmbbm.2013.02.002 23517775

[B54] Komatsu K. Viidik A. (1966). Changes in the fibre arrangement of the rat incisor periodontal ligament in relation to various loading levels *in vitro*. Arch. Oral Biol. 41, 147–159. 10.1016/0003-9969(95)00114-X 8712971

[B55] Kristiansen A. B. Heyreaas K. J. (1989). Micropuncture measurements of interstitial fluid pressure in the rat periodontal ligament. Proc. Finn. Dent. Soc. 85, 295–300. 2699760

[B56] Kurashima K. (1965). Viscoelastic properties of periodontal tissue. Bull. Tokyo Med. Dent. Univ. 36, 148–153.

[B57] Lakes R. S. (1999). Viscoelastic Solids. Boca Raton, FL: CRC Press.

[B58] Lin D. C. Dimitriadis E. K. Horkay F. (2007). Robust strategies for automated AFM force curve analysis-I. Non-adhesive indentation of soft inhomogenous materials. J. Biomech. Eng. 129, 430–440. 10.1115/1.2720924 17536911

[B59] Lindhe J. Karring T. Lang N. P. (2003). Clinical Periodontology and Implant Dentistry. Copenhagen: Blackwell Munksgaard.

[B60] Mahaffy R. E. Shih C. K. MacKintosh F. C. Käs J. (2000). Scanning probe-based frequency-dependent microrheology of polymer gels and biological cells. Phys. Rev. Lett. 85, 880–883. 10.1103/PhysRevLett.85.880 10991422

[B61] Manning L. Rogers B. Jones M. Adams J. D. Fuste J. L. Minne S. C. (2003). Self-oscillating tapping mode atomic force microscopy. Rev. Sci. Instrum. 74, 4220–4222. 10.1063/1.1602935

[B62] Meyer J. Squier C. A. Gerson S. J. (1984). The Structure and Function of Oral Mucosa. Oxford: Pergamon Press.

[B63] Michalakis K. X. Calvani P. Hirayama H. (2012). Biomechanical considerations on tooth-implant supported fixed partial dentures. J. Dent. Biomech. 3:1758736012462025 10.1177/1758736012462025 23255882 PMC3487629

[B64] Mühlemann H. R. (1951). Periodontometry, a method for measuring tooth mobility. Oral. Surg. Oral. Med. Oral. Path 4, 1220–1233. 10.1016/0030-4220(51)90080-1 14882794

[B65] Murphy M. F. Lilley F. Lalor M. J. Crosby S. R. Madden G. Johnston G. (2013). Evaluation of a nonlinear Hertzian-based model reveals prostate cancer cells respond differently to force than normal prostate cells. Microsc. Res. Tech. 76, 36–41. 10.1002/jemt.22132 23070866

[B66] Nanci A. (2013). Ten Cate's Oral Histology. St. Louis, MO: Elsevier Mosby.

[B67] Natali A. N. Pavan P. G. Carniel E. L. Dorow C. (2003). A transversally isotropic elasto-damage constitutive model for the periodontal ligament. Comput. Methods Biomech. Biomed. Engin. 6, 329–336. 10.1080/10255840310001639840 14675953

[B68] Natali A. N. Pavan P. G. Carniel E. L. Dorow C. (2004). Visco-elastic response of the periodontal ligament: an experimental-numerical analysis. Connect. Tissue Res. 45, 222—230. 10.1080/03008200490885742 15763931

[B69] Newcomb G. M. (1981). An ultrastructural study of epithelial specialization at the porcine mucogingival junction. J. Periodont. Res. 16, 51–65. 10.1111/j.1600-0765.1981.tb00949.x 6453972

[B70] Notbohm J. Poon B. Ravichandran G. (2012). Analysis of nanoindentation of soft materials with an atomic force microscope. J. Mater. Res. 27, 229–237. 10.1557/jmr.2011.252

[B71] Picton D. C. (1965). On the part played by the socket on tooth support. Arch. Oral Biol. 6, 945–955. 10.1016/0003-9969(65)90088-9 4960055

[B72] Picton D. C. (1969). The effect of external forces on the periodontium, in Biology of the Periodontium, eds Melcher A. H. Bowen W. H. (New York, NY: Academic Press), 363–419.

[B73] Radmacher M. Tillman R. W. Fritz M. Gaub H. E. (1992). From molecules to cells: imaging soft samples with the atomic force microsope. Science 257, 1900–1905. 10.1126/science.1411505 1411505

[B74] Rahmat B. Hubert P. (2010). Interaction stress measurement using atomic force microscopy: a stepwise discretization method. J. Phys. Chem. C. 114, 15029–15035. 10.1021/jp104993f

[B75] Rogers B. Manning L. Sulchek T. Adams J. D. (2004). Improving tapping mode atomic force microscopy with piezoelectric cantilevers. Utramicroscopy 100, 267–276. 10.1016/j.ultramic.2004.01.016 15231319

[B76] Sader J. E. Chon J. W. M. Mulvaney P. (1999). Calibration of rectangular atomic force microscope cantilevers. Rev. Sci. Instrum. 70, 3967–3969. 10.1063/1.1150021

[B77] Saitoh S. Sasaki K. Nezu T. Taira M. (2010). Viscoelastic behavior of commercially available tissue conditioners under compression. Dent. Mater. J. 29, 461–468. 10.4012/dmj.2009-130 20668361

[B78] Sims M. (1995). The morphology of the vasculature of the periodontal ligament, in The Periodontal Ligament in Health and Disease, eds Berkovitz B. K. B. Moxham B. J. Newman H. N. (London: Mosby-Wolfe), 107–20.

[B79] Slavkin H. C. Bavetta L. A. (1972). Developmental Aspects of Oral Biology. New York, NY: Academic Press.

[B80] Sloan P. Carter D. H. (1995). Structural organization of the fibres of the periodontal ligament, in The Periodontal Ligament in Health and Disease, eds Berkovitz B. K. B. Moxham B. J. Newman H. N. (London: Mosby-Wolfe).

[B81] Stefanadis C. Dernellis J. Tsiamis E. Diamantopoulos L. Michaelides A. Toutouzas P. (2000). Assessment of aortic line of elasticity using polynomial regression analysis. Circulation 101, 1819–1825. 10.1161/01.CIR.101.15.1819 10769283

[B82] Stolz M. Gottardi R. Raitteri R. Miot S. Martin I. Imer R.. (2009). Early detection of aging cartilageand osteoarthritis in mice and patient samples using atomic force microscopy. Nat. Nanotechnol. 4, 186–192. 10.1038/nnano.2008.410 19265849

[B83] Stolz M. Raiteri R. Daniels A. U. VanLandingham M. R. Baschong W. Aebi U. (2004). Dynamic elastic modulus of porcine articular cartilage determined at two different levels of tissue organization by indentation-type atomic force microscopy. Biophys. J. 86, 3269–3283. 10.1016/S0006-3495(04)74375-1 15111440 PMC1304192

[B84] Sweigart M. A. Zhu C. F. Burt D. M. DeHoll P. D. Agrawal C. M. Clanton T. O.. (2004). Intraspecies and interspecies comparison of the compressive properties of the medial meniscus. Ann. Biomed. Eng. 32, 1569–1579. 10.1114/B:ABME.0000049040.70767.5c 15636116

[B85] Tao N. J. Lindsay S. M. Lees S. (1992). Measuring the microelastic properties of biological materials. Biophys. J. 63, 1165–1169. 10.1016/S0006-3495(92)81692-2 1420932 PMC1262253

[B86] Thavarajah R. Mudimbaimannar V. K. Elizabeth J. Rao U. K. Ranganatham K. (2012). Chemical and physical basics of routine formaldehyde fixation. J. Oral Maxillofac. Pathol. 16, 400–405. 10.4103/0973-029X.102496 23248474 PMC3519217

[B87] Trickey W. R. Baaijens F. P. Laursen T. A. Alexopoulos L. G. Guilak F. (2006). Determination of the Poisson's ratio of the cell: recovery properties of chondrocytes after release from complete micropipette aspiration. J. Biomech. 39, 78–87. 10.1016/j.jbiomech.2004.11.006 16271590

[B88] Van Loocke M. Lyons C. G. Simms C. K. (2006). A validated model of passive muscle in compression. J. Biomech. 39, 2999–3009. 10.1016/j.jbiomech.2005.10.016 16313914

[B89] Van Loocke M. Lyons C. G. Simms C. K. (2008). Viscoelastic properties of skeletal muscle in compression: stress-relaxation behavior and constitutive modelling. J. Biomech. 41, 1555–1566. 10.1016/j.jbiomech.2008.02.007 18396290

[B90] Villegas D. F. Maes J. A. Magee S. D. Donahue T. L. (2007). Failure properties and strain distribution analysis of meniscal attachments. J. Biomech. 40, 2655–2662. 10.1016/j.jbiomech.2007.01.015 17359982

[B91] Vinckier A. Semenza G. (1998). Measuring elasticity of biological materials by atomic force microscopy. FEBS Lett. 430, 12–16. 10.1016/S0014-5793(98)00592-4 9678586

[B92] Weisenhorn A. L. Kasas S. Solletti J. M. Khorsandi M. Gotzos V. Romer D. U. (1993a). Deformation observed on soft surfaces with an AFM. Proc. SPIE 1855 26–34. 10.1117/12.146382

[B93] Weisenhorn A. L. Khorsandi M. Kasas S. Gotzos V. Butt H. J. (1993b). Deformation and height anomaly of soft surfaces studied with an AFM. Nanotechnology 4, 106–113. 10.1088/0957-4484/4/2/006

[B94] Weisenhorn A. L. Maivald P. Butt H. J. Hansma P. K. (1992). Measuring adhesion, attraction, and repulsion between surfaces in liquids with an atomic-force microscope. Phys. Rev. B. Condensed. Matt. 45, 11226–11232. 10.1103/PhysRevB.45.11226 10001046

[B95] Weiss J. A. Gardiner J. C. Bonifasi-Lista C. (2002). Ligament material behavior is nonlinear, viscoelastic and rate-independent under shear loading. J. Biomech. 35, 943–950. 10.1016/S0021-9290(02)00041-6 12052396

[B96] Wills D. J. Picton D. C. (1978). Changes in the mobility and resting position of incisor teeth in macaque monkeys. Arch. Oral. Biol. 23, 225–229. 10.1016/0003-9969(78)90221-2 99129

[B97] Wills D. J. Picton D. C. Davies W. I. R. (1976). A study of the fluid systems of the periodontium in macaque monkeys. Arch. Oral. Biol. 21, 175–185. 10.1016/0003-9969(76)90127-8 820316

[B98] Yang F. (1998). Indentation of an incompressible elastic film. Mech. Mater. 30, 275–286. 10.1016/S0167-6636(98)00035-0

[B99] Yang F. (2006). Effect of adhesion energy on the contact stiffness in nanoindentation. J. Mater. Res. 21, 2683–2688. 10.1557/jmr.2006.0331

[B100] Yang S. Zhang Y. W. Zeng K. Y. (2004). Analysis of nanoidentation creep for polymeric materials. J. Appl. Phys. 95, 3655–3666. 10.1063/1.1651341

[B101] Yin L. Elliot D. M. (2004). A biphasic and transversely isotropic mechanical model for tendon: application to mouse tail fascicles in uniaxial tension. J. Biomech. 37, 907–916. 10.1016/j.jbiomech.2003.10.007 15111078

[B102] Zhang Y. L. Han M. L. Vidyalakshmi J. Shee C. Y. Ang W. T. (2009). Automatic control of mechanical forces acting on cell biomembranes using a vision-guided microrobotic system in computer microscopy. J. Microsc. 236, 70–78. 10.1111/j.1365-2818.2009.03209.x 19772538

